# Efficacy of different concentrations of sodium hypochlorite and chlorhexidine in disinfection of contaminated Resilon cones

**DOI:** 10.4317/medoral.17467

**Published:** 2011-12-06

**Authors:** Vahid Zand, Amin Salem-Milani, Shahriar Shahi, Mohammad T. Akhi, Siamak Vazifekhah

**Affiliations:** 1 DDS, MSc, Assistant Professor, Department of Endodontics, Faculty of Dentistry, Tabriz University of Medical Sciences, Tabriz, Iran; 2 DDS, MSc, Assistant Professor, Dental and periodontal disease research center, Faculty of Dentistry, Tabriz University of Medical Sciences, Tabriz, Iran; 3 DDS,MSc, Associate Professor, Dental and periodontal disease research center, Faculty of Dentistry, Tabriz University of Medical Sciences, Tabriz, Iran; 4 PhD, Associate professor of Medical Microbiology Department, Tabriz University of Medical Sciences; 5 DDS, Dentist

## Abstract

Objectives: The aim of this study was to evaluate the effectiveness of different concentrations of Chlorhexidine (CHX) and sodium hypochlorite (NaOCl) in disinfecting contaminated Resilon cones within one minute. Study design: Fifty Resilon cones were divided into seven experimental groups and three control groups of 5 cones each. The cones of experimental groups were contaminated with *Entrococcus faecalis* and subsequently disinfected with different concentrations of NaOCl or CHX. The cones were then transferred into glass tubes containing thioglycollate media and incubated for 7 days. The tubes were examined for turbidity every 24 hours, and if bacterial growth occurred, samples were plated, incubated, gram stained and observed under microscope to confirm *E.faecalis* growth. Negative, positive, and washing control groups were also used. Results: All the positive and washing control showed profound *E.faecalis* growth. All the cones disinfected with CHX showed bacterial growth; however, no *E.faecalis* growth occurred in any samples disinfected with NaOCl. Conclusion: Sodium hypochlorite, at concentrations of 0.5 to 5.25%, is an effective agent for disinfection of contaminated Resilon cones within one minute; however, chlorhexidine is unable to disinfect Resilon cones during one-minute exposure.

** Key words:** Chlorhexidine, sodium hypochlorite, resilon, entrococcus faecalis.

## Introduction

Microorganisms are the main etiologic factor of pulp and periapical pathosis (1). Thus, the primary goal of endodontic treatment is the elimination of microorganisms from infected root canals and prevention of their re-entry. For this purpose, every phase of endodontic therapy should be performed under aseptic conditions.

Resilon (Pentron Clinical Technologies LLC, Wallingford, CT) is a thermoplastic polycaprolactone-based root canal material which is similar to gutta-percha in many properties such as size, color, and handling (2). Resilon is used with a resin-based sealer, Epiphany, and according to the manufacturer, forms a “monoblock” within the canal. Several studies have shown that this “monoblock” has less microleakage than gutta-percha fillings and strengthens the tooth structure (2,3). Gutta-percha and Resilon cones are produced under aseptic conditions; however, they can be contaminated by aerosols and physical sources during storage (4-6). Even if the cones are carefully removed from the package, they may become infected with microorganisms by handling (6-8). Gutta-percha and Resilon cones can not be disinfected with routine heat sterilization methods; therefore, an alternative rapid disinfection approach is desirable. Different chemicals have been proposed for rapid chemical disinfection of gutta-percha cones. Sodium hypochlorite (NaOCl), as the most common irrigating solution, has been used in several studies to disinfect contaminated gutta-percha or Resilon cones (7-12). The results were dependent on the concentration of NaOCl and exposure time (5). Chlorhexidine (CHX), as another endodontic irrigant, is a broad-range disinfectant which is effective against most bacteria and yeasts found in endodontic infections. CHX has also been used in some studies to disinfect gutta-percha or Resilon cones with contradictory results regarding the most proper time and concentration of CHX or NaOCl to disinfect contaminated Resilon cones. Dumani et al. (12) found that 2% CHX is not effective in disinfection of Resilon cones at one-minute exposure. However, Gomes and Royal et al. showed that 2% CHX is an effective agent in eliminating *Entrococcus faecalis* even within 15 seconds (5,7). All these studies have used 2% CHX, and there is no published study on the efficacy of the different concentrations of CHX. Because of this controversy, the present study was designed to evaluate the effectiveness of different concentrations of NaOCl and CHX in rapid disinfection of contaminated Resilon cones. 

## Material and methods

**Figure 1:**
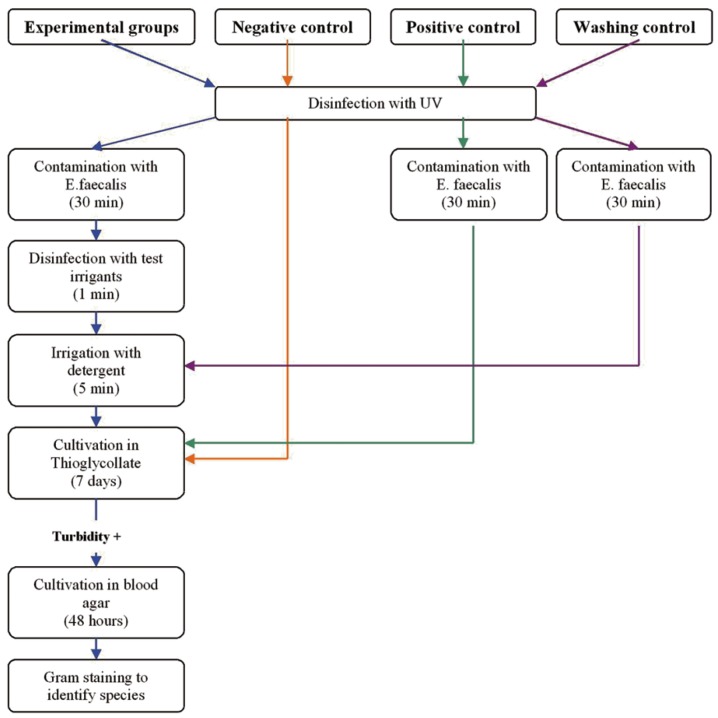
Diagram representing the process of the study.

Fifty Resilon cones (Pentron Clinical Technologies LLC, Wallingford, CT) were divided into seven experimental groups and three control groups of 5 cones each. The method of the experiment has been summarized in (Fig. 1). The cones were disinfected with UV light (250 microW/cm2) for 20 minutes. The experimental cones were immersed in 20 ml of microbial suspension of 108 CFU/ml *Entrococcus faecalis* (*E. faecalis*) ATCC29212 in Trypticase soy broth for 30 minutes and then transferred into Petri dishes and allowed to dry for 10 minutes at room temperature. Then, cones of each experimental group were immersed in separate Petri dish containing 20 ml of the disinfectants with different concentrations for one minute. The experimental groups were as follows: group1: 0.5% NaOCl, group 2: 1% NaOCl, group 3: 2.5% NaOCl, group 4: 5.25% NaOCl, group 5: 0.5% CHX, group 6: 1% CHX, group 7: 2% CHX. The cones of experimental groups were then immersed in detergent (3% Tween 80 plus 5% sodium thiosulfate) for 5 minutes and washed with 10 ml of sterile distilled water. The cones were separately transferred into glass tubes containing 10 ml of thioglycollate and incubated at 37 ºC for 7 days. A blinded observer examined the tubes every 24 hours for turbidity which indicated bacterial growth in the tubes. At the end of 7 days, in the case of bacterial growth, 0.1 ml of thioglycollate was inoculated into 10% blood agar and incubated at 37 ºC for 48 hours. The bacterial colonies in blood agar plates were the signs of bacterial growth. Gram staining and observation under light microscope were used to confirm that the bacteria were *E. faecalis*.

Three control groups were used as follows: In negative control, the cones were disinfected under UV and directly transferred into thioglycollate and examined for bacterial growth to check the primary sterility of the samples. In positive control, the cones were disinfected under UV, contaminated with *E. faecalis* similar to experimental groups and then transferred into thioglycollate and examined for bacterial growth to check the accuracy of contamination process. In the washing control group, the process was the same as experimental groups except that exposure to disinfectants was not carried out. 

The number of growth positive samples in experimental or control groups were recorded and analyzed with descriptive statistical methods using SPSS software for windows (SPSS Inc, Chicago, IL).

## Results

All the positive and washing control showed heavy turbidity and *E. faecalis* growth was confirmed by culturing and gram staining. No bacterial growth occurred in any negative control samples.

All the cones disinfected with various concentrations of CHX showed *E. faecalis* growth. However, no *E. faecalis* growth occurred in any samples disinfected with different concentrations of NaOCl ( Table 1).

**Table 1:**
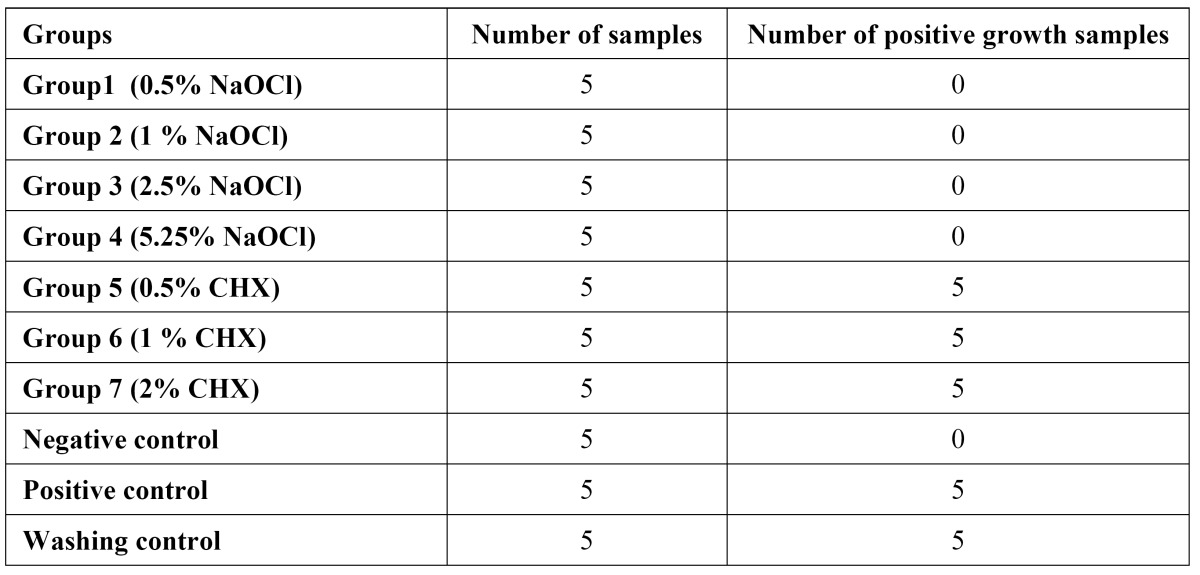
Microbial growth of *E.faecalis* after disinfection with different concentrations of sodium hypochlorite (NaOCl) and Chlorhexidine (CHX).

## Discussion

One of the causes of root canal reinfection is the use of contaminated root canal fillings (7). Gutta-percha or Resilon cones are manufactured under aseptic conditions; however, contamination may occur after removal from the package as a result of exposure to environment and by handling (4-6,8). Therefore, rapid disinfection of Resilon or gutta-percha cones prior to obturation is desirable. A chemical disinfection is appropriate because heat sterilization alters the properties of cones. NaOCl and CHX are two common endodontic irrigants and have been used in some studies with varying concentrations for chemical disinfection of root canal filling materials (5,7,9-12). 

 E.faecalis was chosen for this study because this species has been shown to be a predominant bacterial species in resistant endo-dontic cases (13,14). *E. faecalis* has also been used in many studies on the efficacy of endodontic irrigants (15,16).

In our study, NaOCl with 0.5%, 1%, 2.5% or 5.25% concentrations was effective in decontamination of Resilon cones. This is in accordance with the results of other studies (5,7,10-12); however, our study showed that chlorhexidine is not effective in decontamination of Resilon cones within one minute. Similarly, Dumani et al. (12) found that 1-min treatment with 2% CHX is not sufficient to disinfect the Resilon cones, and it is effective only after 5-min treatment. However, Royal et al. (7) showed that one-min treatment with 2% CHX is sufficient to disinfect contaminated gutta-percha or Resilon cones. Both of these studies used *E. faecalis* as test species and followed similar process except that Royal et al. (7) used Resilon pellets instead of cones because of easy handling, and the bacterial exposure time was 5 min in contrast to our study which used 30 min exposure. Regardless of these differences in methods and materials, these contradictory results need more investigation. 

Gomes et al. (5) also showed that even 1% CHX is effective within 15 seconds in decontamination of gutta-percha cones. It’s difficult to compare the results of our study with the results of studies on gutta-percha because Resilon and gutta-percha have different chemical composition and surface texture (12). In addition, unlike Resilon, gutta-percha has antimicrobial property which may be an explanation for the less time required to disinfect gutta-percha cones by CHX than Resilon cones (17,18). 

We recommend that this study be repeated with other bacterial species, different forms of Resilon (cones or pellets), and varying bacterial exposure times. Blindness of the examiners is another important factor that may influence the results of the study and is recommended to be taken into consideration in future studies. 

## Conclusion

Sodium hypochlorite, at concentrations of 0.5 to 5.25%, is an effective agent for disinfection of contaminated Resilon cones within one minute; however, chlorhexidine is unable to disinfect Resilon cones during 1-min exposure.
